# Mass Spectrometric and Synchrotron Radiation based techniques for the identification and distribution of painting materials in samples from paints of Josep Maria Sert

**DOI:** 10.1186/1752-153X-6-45

**Published:** 2012-05-22

**Authors:** Anna Lluveras-Tenorio, Alessia Andreotti, Ilaria Bonaduce, Sarah Boularand, Marine Cotte, Josep Roqué, Maria Perla Colombini, Marius Vendrell-Saz

**Affiliations:** 1 Departament de Cristal.lografia, Mineralogia I Dipòsits Minerals, University of Barcelona, C/Marti i Franquès S/N, 08015, Barcelona, Spain; 2 Dipartimento di Chimica e Chimica Industriale, University of Pisa, Via Risorgimento 35, 56126, Pisa, Italy; 3 European Synchrotron Radiation Facility, 6 rue Jules Horowitz, F-38000, Grenoble, France; 4 Centre de Recherche et de Restauration des Musées de France, CNRS UMR171, Palais du Louvre, Porte des Lions, 14 Quai François Mitterrand, F-75001, Paris, France; 5 Science Division, Diamond Light Source Ltd, Harwell Science and Innovation Campus, Oxon, OX11 0DE, United Kingdom

**Keywords:** GC/MS, Mapping, FTIR, Synchrotron radiation, XRD, Paintings

## Abstract

**Background:**

Establishing the distribution of materials in paintings and that of their degradation products by imaging techniques is fundamental to understand the painting technique and can improve our knowledge on the conservation status of the painting. The combined use of chromatographic-mass spectrometric techniques, such as GC/MS or Py/GC/MS, and the chemical mapping of functional groups by imaging SR FTIR in transmission mode on thin sections and SR XRD line scans will be presented as a suitable approach to have a detailed characterisation of the materials in a paint sample, assuring their localisation in the sample build-up. This analytical approach has been used to study samples from Catalan paintings by Josep Maria Sert y Badía (20^th^ century), a muralist achieving international recognition whose canvases adorned international buildings.

**Results:**

The pigments used by the painter as well as the organic materials used as binders and varnishes could be identified by means of conventional techniques. The distribution of these materials by means of Synchrotron Radiation based techniques allowed to establish the mixtures used by the painter depending on the purpose.

**Conclusions:**

Results show the suitability of the combined use of SR μFTIR and SR μXRD mapping and conventional techniques to unequivocally identify all the materials present in the sample and their localization in the sample build-up. This kind of approach becomes indispensable to solve the challenge of micro heterogeneous samples. The complementary interpretation of the data obtained with all the different techniques allowed the characterization of both organic and inorganic materials in the samples layer by layer as well as to establish the painting techniques used by Sert in the works-of-art under study.

## Background

Paintings are complex systems due to the fact that they are multi-material, multi-layered. The painting technique is thus determined not only by the knowledge of which materials constitute a work of art but also by determining their distribution, layer by layer.

The use of conventional techniques, namely optical (OM), scanning electron microscopy coupled with Electron Dispersive Spectroscopy (SEM-EDS), micro Fourier Transform Infrared Spectroscopy (μFTIR), Raman spectroscopy, Gas Chromatography/Mass Spectrometry (GC/MS) and Pyrolysis/Gas Chromatrography/Mass spectrometry (Py/GC/MS) can provide a detailed and almost complete characterization of the materials present in a painting.

However a complete analysis of the painting requires both in plane and in depth information and an imaging of organic and inorganic materials of the paint cross sections is fundamental for an in depth characterization of painting systems, allowing the elemental and molecular heterogeneities to be resolved both within and between layers [1-4].

In this paper a multi-analytical approach for the characterization of organic and inorganic materials in paint micro samples is shown. OM and SEM-EDS were used for the morphological characterization of the samples. Conventional Fourier Transform Infrared Spectroscopy (FTIR), analytical Pyrolisis in the presence of hexamethyldisilazane coupled on line with gas chromatography/mass spectrometry analysis (Py/GC/MS) [1,5] as well as a GC/MS analytical procedure for the identification of lipids, waxes, proteins, and resinous materials in the same microsample was then used for the identification of organic materials and their degradation products in the bulk sample [6] were used to characterize the materials (organic and some inorganic) in the bulk of the samples. Finally, Synchrotron Radiation (SR) micro FTIR in transmission mode allowed the establishment of the chemical images of the functional groups in a thin section highlighting the distribution of these materials both in depth and along the sample [7-13]. Transmission has been chosen due to its higher spectra quality, easier interpretation and wider database. To complement the characterization of pigments, dryers and fillers, Synchrotron Radiation micro X-Ray Diffraction (SR XRD) line scans in transmission mode was used to establish the crystalline phases present [14,15].

This work describes the results obtained by the application of the above mentioned multi- analytical approach to three samples from Josep Maria Sert’s paintings in order to establish the painting technique used by the painter. Josep Maria Sert i Badia (1876–1945) was one of the most famous Catalan muralists of the beginning of the 20^th^ achieving international recognition. His big size canvases adorned the walls of such buildings as the assembly hall of the League of Nations (Geneva), the RCA Building in Rockefeller Centre and the Waldorf-Astoria Hotel (both in New York City). Sert’s painting technique is of particular interest because the painter’s work changed from polychrome and decorative mural paintings in his former works to almost monochrome paintings (sepia, gilded and silvery tonalities). In the occasion of the publication of a book on Sert paintings in the city of Vic (Barcelona) some of his paintings have been studied [16]. A total of seven samples were collected from six canvases painted between 1906–1945. Sampled canvases were the “Fight Between Jacob and the Angel” (1906), the winter and spring panels of “The Fourth Seasons” (1917–1920), “Heliodor Expelled from the Temple” (1920) and the central panel of the painting “Crucifixion” (1945).

On the basis of the results obtained on the preliminary analyses of the seven samples by SEM-EDS, FTIR and Py/GC/MS (the whole body of data are provided as supplementary information) it was decided to proceed with SR μFTIR mapping and SR μXRD linear scan on three samples that were representative of the two different painting techniques used by the painter. This paper presents the results obtained for the three chosen samples, and discusses the data in order to reconstruct the painting technique.

### Experimental section

#### Reagents

For the chromatographic technique all the solvents used were Baker HPLC grade. Hexadecane, tridecanoic acid and norleucine, used as internal standards, hexamethyldisilazane (HMDS), and *N,O*-bis(trimethylsilyl)trifluoroacetamide (BSTFA) containing 1% trimethylchlorosilane were purchased from Sigma (Milan, Italy). *N*-*tert*-Butyldimethylsilyl-*N*-methyltrifluoroacetamide (MTBSTFA) with 1% trimethylchlorosilane was from Fluka (USA). All reagents and chemicals were used without any further purification. Standard solutions of amino acids in hydrochloric acid (0.1 M), containing 12.5 μmol/mL of proline and hydroxyproline, 1.25 μmol/mL of cysteine and 2.5 μmol/mL of aspartic acid, glutamic acid, alanine, arginine, phenylalanine, glycine, hydroxylysine, isoleucine, histidine, leucine, lysine, methionine, serine, tyrosine, threonine, and valine was purchased from Sigma-Aldrich (USA). A solution containing lauric acid, suberic acid, azelaic acid, myristic acid, sebacic acid, palmitic acid, oleic acid, stearic acid (all purchased from Sigma-Aldrich, USA) in the range of 2–3 μ/g was prepared in isooctane and stored at 4°C.

A polyester resin polymerised by a peroxy organic hardener (Cronolite E.I, Plastiform, Spain) was used for the cross-section preparation. The epoxy resin used for the SR FTIR slices was purchased at Plastiform, Spain.

### Apparatus and analytical procedure

Stereo microscope Nikon SMZ 1500 (Izasa S.A., Barcelona, Spain)

Nikon Eclipse LV 100 PDL polarizing microscope equipped with a Nikon Digital Camera DMX 1200 F (Izasa S.A., Barcelona, Spain).

Scanning Electron Microscope (SEM) JEOL (Tokyo, Japan) JSM-840 (secondary and backscattered electron detection) coupled with an Energy Dispersive X–ray Spectroscopy (EDS) facility LINK AN 10000 microanalyser. The acceleration voltage used was 20 keV. EDS mappings were collected by using a Cambridge Leica Stereoscan S-360 coupled with INCA Energy Sèrie 200 microanalyser (Oxford Instruments). Conditions were as follow: filament 2,8 A, probe 3 nA and EHT 20 kV.

Bomem MB-120 Fourier Transform Infrared Spectrometer equipped with a DTGS detector. The spectra are the sum of 30 scans collected from 4000 to 350 cm^-1^ at a resolution of 4 cm^-1^ when working with the diamond cell.

Bomem MB-120 Fourier Transform Infrared Spectrometer, equipped with a Spectra-Tech Analytical Plan microscope, was used with the diamond cell, as a sample holder. The spectrometer has a KBr beamsplitter and a Globar source. The microscope has its own mercury cadmium telluride (MCT) detector refrigerated with liquid nitrogen. Spectrum was recorded between 4000 and 720 cm^-1^ with a resolution of 4 cm^-1^ and an accumulation of 100 scans.

Pyroprobe CDS Analytical Inc. 5000 Series (Oxford, USA). It was operating with an initial temperature of 50°C, up to 550°C at 20°C/ms, then isothermal for 20 sec. (probe run time 0.33 min). The pyrolyser was coupled on-line with the injection port of a 6890 N GC System Gas Chromatograph (Agilent Technologies, Palo Alto, CA, USA), coupled with a 5973 Mass Selective Detector (Agilent Technologies, Palo Alto, CA, USA) single quadrupole mass spectrometer, equipped with split/splitless injector. The interface Py/GC temperature was 180°C, the transfer line 300°C, the valve oven 290°C. The mass spectrometer was operating in the electron impact (EI) positive mode (70 eV). A few μg of the samples admixed with 2 μl of hexamethyldisilazane were inserted into a quartz tube. Detailed working conditions are published elsewhere [17].

A 6890 N GC System Gas Chromatograph (Agilent Technologies, Palo Alto, CA, USA), coupled with a 5975 Mass Selective Detector (Agilent Technologies, Palo Alto, CA, USA) single quadrupole mass spectrometer, equipped with a PTV injector was used. The mass spectrometer was operating in the electron impact (EI) positive mode (70 eV). The MS transfer line temperature was 280°C; the MS ion source temperature was kept at 230°C; and the MS quadrupole temperature was at 180°C. This instrument was used for the analysis of samples processed with the combined analytical procedure for the simultaneous identification of glycerolipids, proteinaceous materials, plant and animal resins, and natural waxes in the same micro sample. The procedure is based on a sample multi step chemical pre-treatment (solvent extractions and microwave-assisted chemolysis) that is able to separate the various organic components into three different fractions: amino acid, acidic and neutral fractions. The detailed operating conditions, and the analytical procedure are published elsewhere [6].

Microwave oven model MLS-1200 MEGA Milestone (FKV, Sorisole (BG,) Italy). Acidic hydrolysis conditions were : power 250 W for 10 min; power 500 W for 30 min in the vapor phase with 30 mL of 6 N HCl at 160°C for 40 min. Saponification conditions were: power 200 W with 300μL of KOH_ETOH_ 10% wt at 80°C for 60 min [6].

Synchrotron radiation Fourier transform infrared microspectroscopy (SR FTIR) in transmision mode was performed at the end-station ID21 at the European Radiation Synchrotron Facility (ESRF, Grenoble, France). The microscope is a Continuμm (Thermo) coupled with a Nexus Spectrometer (Thermo). The detector is a 50 μm MCT. Maps were recorded using 4 microns step and 40 scans for each spectrum. Beam spot and resolution were fixed at 8 × 8 μm² and 8 cm^-1^, respectively. In all cases the aperture and the step size chosen generate overlapping areas in order to increase the resolution of the components [18].

Microtome Ultracut E with a tungsten knife for slices of less than 12 μm was used.

SR XRD (Synchrotron radiation X Ray diffraction) patterns in transmission mode were acquired at the beamline ID18F of the ESRF. A focal spot of 2,3 μm in the vertical direction and 11 μm in the horizontal direction was chosen with steps of 2 μm in the vertical direction. A wavelength of 0,443Å (28 keV) was selected and the acquisition time was 20 seconds per pattern. The diffraction signal was recorded in transmission by means of a 2-dimensional CCD-based X-ray detector. The cross-section was placed into the focused beam with the paint layers oriented horizontally. The sample preparation for transmission XRD experiments has been already reported in previous works. It consists in an embedding of the fragment in polyester resin polymerised by a peroxo organic hardener under low humidity conditions and sectioned with a diamond saw of thickness 0,1 mm into a 200 microns thick slice [13,19]. Patterns were fitted with the ESRF FIT2D package software [20].

### Samples

**Figure 1:**
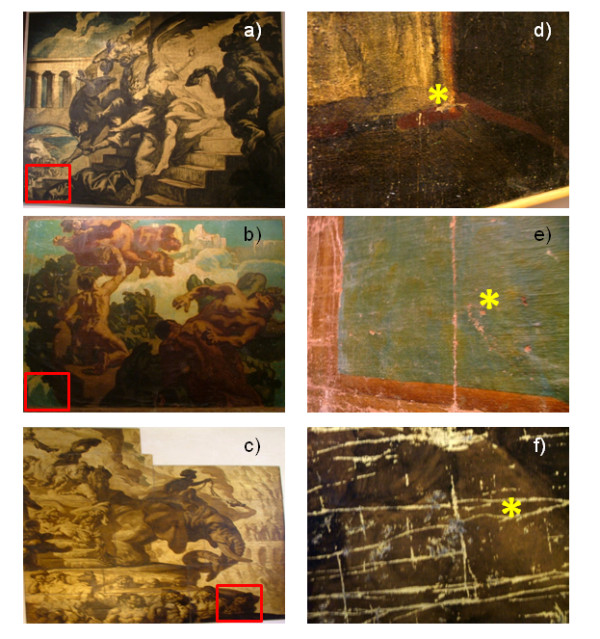
**Sampled canvases: a) “Heliodor Expelled from the Temple”, b) “Fight Between Jacob and the Angel”, c) “In Honor of the East”; the square evidences the area sampled showed in d), e) and f), respectively.** The * marks the specific sampling point corresponding to samples VIC 2 (**d**), VIC 5(**e**) and VIC 7(**f**).

The samples chosen for the application of the multi analytical approach, the canvases of provenience and the sampling point description are summarized in Table 1. A picture of the sampled canvases is presented in Figure 1. Samples were always taken from nicks of the canvases trying to minimize the damage to the paintings. The selection was made in order to represent the different techniques applied by Sert, from his classical and standard oil paintings (sample VIC 5) to the multilayered metallic application with interposed varnishes and colored layers (samples VIC 2 and VIC 7).

**Table 1:** Description of the paint samples

**Sample name**	**Painting**	**Year**	**Sampling area**
VIC 2	“Heliodor Expelled from the Temple”	1920	Black line next to a red one
VIC 5	“Fight Between Jacob and the Angel”	1906	green colour
VIC 7	“In Honor of the East”	1926	Gilded area

## Results and discussion

### Morphological characterisation

**Figure 2:**
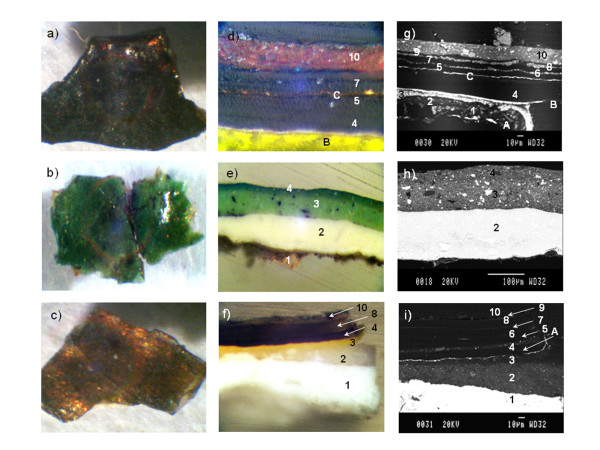
**Stereomicroscope images of the surfaces of samples a) VIC 2; b) VIC 5 and c) VIC 7; Optical microscopic image of the polished cross-section d) VIC 2; e) VIC 5; f) VIC 7; scanning electron microscope images of the cross-section g) VIC 2; h) VIC 5; i) VIC 7.** Paint layers are indicated with numbers while metallic layers are indicated with A, B, C.

Figure 2 shows the stereomicroscope images of the samples surface and the optical and electron microscope images of their cross-sections. Table 2 summarizes the results of the morphological characterization of the samples. The composition of the metallic layers obtained with the EDS is reported as well, while the elemental composition of the other layers is summarized in Table 3. Table 3 summarizes the inorganic compounds identified and their distribution in the sample layers with the different techniques discussed below.

**Table 2:** Summary of the morphological characterization of the samples

**Sample**	**Appearance of the surface**	**Total number of layers**	**Thickness (min-max /μm)**	**Number of highly pigmented layers**	**Number of mainly organic layers**	**Number of metallic layers**	**Metallic leaves**
VIC 2	Dark red	13	2-20	6	4	3	AuCu-Zn
VIC 5	Green	4	25-100	4	0	0	-
VIC 7	golden	11	1-100	6	4	1	Au

**Table 3:** Results of the different techniques for VIC 2, VIC 5 and VIC 7 and inorganic compounds identified

**Sample**	**Layer number**	**EDS results**	**FTIR bands**	**XRD crystalline phases**	**Inorganic compounds identified**
VIC 2	10	Al, S, Si, P, Ca, Cr,Ba,Na, Fe	550, 456 1420, 876 1586 1318 743, 723	-	Iron oxide calcite zinc compound calcium oxalates Quartz
	9	S, Al, P, Ca, Si, Na, K> Zn, Fe	2012 2092		Bone black Prussian blue
	8	Cl	-		-
	7	Cl	-		-
	6	Al, S, Si, P, Ca, Cr,Ba,Na > Fe			-
	5	Cl			
	A	Au >>> Ag, Cu			Gold
	4	Cl			-
	B	Cu, Zn			brass
	3	Pb, Cr, Ba	1170,1112,1072, 984, 635, 608 873,820		Barium sulphate Lead chrome yellow (PbCrO_4_)
	2	S, Ba, Pb, Cr			
	1	Fe, Mn, Pb			Clay? Iron oxide?
	C	Cu, Zn			brass
VIC 5	4	Sr, Pb, Zn, Cr, Cd, Fe, Al, Si	923, 909, 842 2092 1400, 680 3698, 3636, 1030, 1008, 916 1585	-	Strontium yellow Prussian blue Lead white Kaolinite Zinc compound
	3				
	2	Pb	3538, 1400, 1047		Lead white
	1	-	-		-
VIC 7	10	Cl		-	
	9	Ca	2012	apatite	Bone black
	8	Cl	-	-	-
	7	-	-	-	-
	6	Cl	-	-	-
	5	P, Ca, Na, Al, Si, Mg	-	Apatite Hydroxyapatite barite	Bone black
	4	Cl	-	-	-
	A	Au	-	Crocoite, Au	Chrome yellow Gold
	3	Zn, P, Ca, Pb	3532, 1400, 837	cerussite hydrocerussite; Zinc oxide; mirabilite	Lead white Zinc white
	2	Zn	-	Zinc oxide;	Zinc white
	1	Pb, Zn	3532,1420, 1052, 837 1524	Cerussite hydrocerussite Lead acetate	Lead white Lead acetate

The morphological characterization of the samples evidenced the differences of the two pictorial techniques identified: two different kinds of stratigraphies and superficial appearances. In particular a first group, represented by sample VIC 5, presented few pictorial layers (mainly a preparation layer together with one or a couple of pigmented layers) with a colored superficial appearance. The second group is represented by samples VIC 2 and 7, which present a dark surface with hints of golden shiny reflects. Their stratigraphies show several layers (more than 10) amongst which there are always at least a metallic layer and an alternation of pigmented and unpigmented layers. It is interesting to notice how thin some of the layers are (1–2 μm).

### Fourier transformed infrared spectroscopy

**Figure 3:**
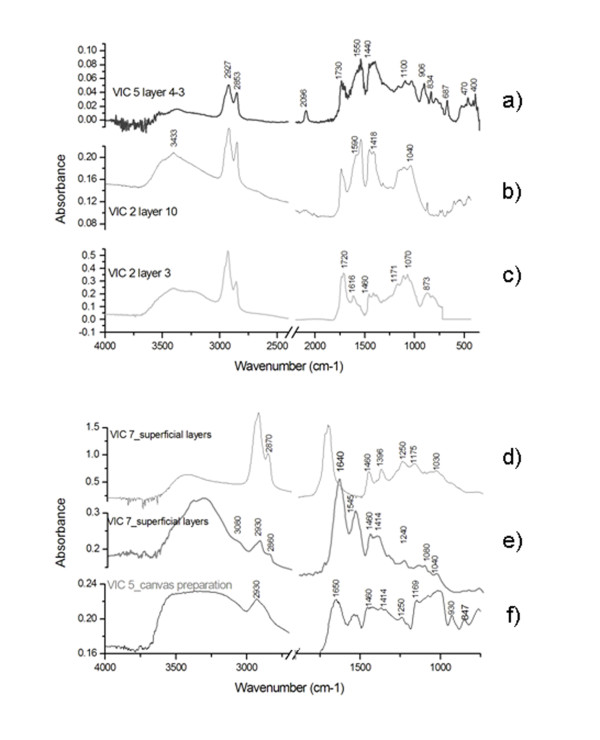
**Conventional FTIR spectra of some of the samples: a) b) and c) representative spectra of pigment layers where oil is detected as binding media; d) natural resin features, e) proteinaceous material bands and f) polysaccharide material.** Spectra have been recorded in transmission mode at a resolution of 4 cm^-1^. VIC 5 and VIC 2 layer 10 are the sum of 30 scans collected from 4000 to 350 cm^-1^ while the rest of the spectra are the sum of 100 scans collected from 4000 to 720 cm^-1^.

Figure 3 shows some spectra representative of the materials identified in the samples VIC 2, VIC 5 and VIC 7. In order to obtain information on the distribution of both organic and inorganic materials, layers were selectively sampled (when possible) using tungsten needles [21] under the stereomicroscope. The identification of the different materials in the sample spectra was made by comparison with reference data [19,22-30].

An oil medium can be identified in the spectra by the presence of the characteristic CH stretching and carbonyl (C = O) stretching bands in Figure 3 a, b and c in the regions, 3000–2800 cm^-1^ and 1750–1650 cm^-1^, respectively [19,23]. In almost all cases the formation of metal carboxylates could be observed in the region 1650–1500 cm^-1^[30]. In the pigmented layers (Figure 3 a,b), carbonyl vibrations are observed at around 1740 cm^-1^ due to esters and the band intensity is lower than that of the metal carboxylate band. This indicates the hydrolysis of the triglycerides and the reaction of the carboxylic acids to metal carboxylates. On the contrary, in the organic layers underneath the metallic leaves(mordant layers) (Figure 3c) the band around 1715 cm^-1^ corresponds to the carboxylic acid moieties [30]. The presence of different kind of pigments and the pigment concentration in the layer, the different coordination states of the carboxylic acid around the metal atom or the nature of the carboxylic acid justify the variations in the frequencies observed for the carboxylates [23,30].

Bands corresponding to inorganic materials (Table 3) such as prussian blue (2092 cm^-1^), lead white (3533, 1410, 1047, 683 cm^-1^) and strontium yellow (923, 909, 842 cm^-1^), calcium oxalates (1640. 1318, 790 cm^-1^), quartz (743, 723 cm^-1^) and iron oxides (550, 456 cm^-1^), chrome yellow (820, 870 cm^-1^) and barium sulphate (1150, 1117, 1085, 635, 610 cm^-1^) can also be easily identified in the spectra shown in Figure 3a, 3b and 3c.

Finally, the spectrum presented in Figure 3d resembles that of a natural resin while the ones presented in Figure 3e and f present the characteristic features of a proteinaceous and polysaccharide material detected in VIC 7 and VIC 5, respectively.

Table 4 summarizes all the data obtained for samples VIC 2, VIC 5 and VIC 7 indicating the position of the compounds in the sample layers when possible.

**Table 4:** FTIR results for the paint samples

**Sample**	**Layer**	**Lipid material**	**Natural resin**	**Proteinaceous material**	**Polysaccharide materials**	**Inorganic materials**
VIC 2	Superficial layers	+	+	-	-	Calcite (CaCO_3_) Iron oxide Calcium oxalates (CaC_2_O_4_) Quartz (SiO_2_)
	Under metallic leave	+		-	-	Barium sulphate (BaSO_4_) Chrome yellow (PbCrO_4_) Metal Carboxylates
VIC 5	Pigment layers on top	+	-	-	-	strontium yellow (SrCrO_4_) Prussian blue (Fe[Fe(CN)_6_]_3_) Clay (kaolinite) Lead white (2 PbCO_3_· Pb(OH)_2_) Calcium oxalates (CaC_2_O_4_) Metal Carboxylates
	Preparation	+	-	-	-	Lead white (2 PbCO_3_· Pb(OH)_2_)
	Canvas prep	-	-	-	+	-
VIC 7	Superficial layers	-	+	+	-	-
	Mordent	+	-	-	-	Chrome yellow (PbCrO_4_) Barium sulfate (BaSO_4_)
	Preparation	+	-	-	-	Lead white (2 PbCO_3_· Pb(OH)_2_)

Information on the layers is given when possible

### Pyrolysis/gas chromatography/mass spectrometry

The organic materials contained in the multi layered samples are summarized in Table 5, and particularly, it could be assessed that:

**Table 5:** Summary of results from the Py/GC/MS analysis

Sample	Drying oil	Pinaceae resin	Shellac	Saccharide material	Proteinaceous material
VIC 2	+	+	+	+	-
VIC 5	+	-	-	+	+ (egg)
VIC 7	+	+	+	+	-

**Figure 4:**
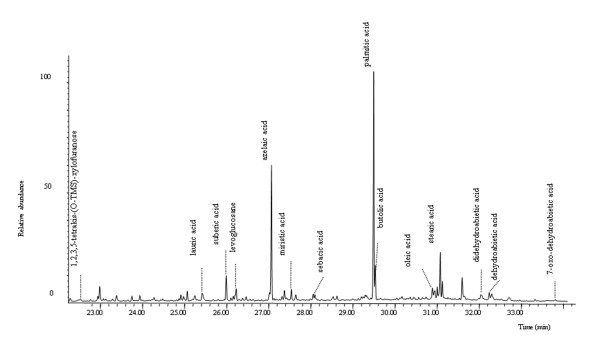
**Pyrogram of sample VIC 2.** Peaks are labelled with the compounds identified.

- samples VIC 2 and 7 (Figure 4 shows the pyrograms of the sample VIC 2) are characterised by the presence of monocarboxylic acids and a relatively high content of dicarboxilic acids attributable to a siccative oil [5]. Dehydroabietic acid together with didehydroabietic and 7-oxo dehydroabietic acid are markers of a Pinaceae resin while butolic acid is indicative of shellac [18,31]. Markers of a well preserved *Pinaceae* resin such as pimaric acid, sandaracopimaric acid or isopimaric acid were not present in any of the samples. Levoglucosane, pyrolysis product of a glucose containing material, and xylofuranose, characteristic pyrolysis product of natural gums (fruit tree, tragacanth, arabic gums or their mixtures) [5], have been found, suggesting the presence of a mixture of polysaccharide materials.

- VIC 5 sample presents a pyrogram characterized by relatively high amounts of monocarboxylic acids, being palmitic and stearic acids the most abundant, and low amounts of dicarboxylic acids. This profile suggests the presence of a siccative oil together with a non drying lipid material. This non drying fat has been identified as egg on the basis of the presence of traces of hexadecanonitrile and octadecanonitrile (markers of egg) in the pyrogram. Markers of both plant and animal resins were absent. The markers of polysaccharide materials in sample VIC 5 presented a similar profile to samples VIC 2–7, indicating that also in this case a mixture of polysaccharide binders is present [5].

### Gas chromatography/mass spectrometry

The analysis of the amino acidic fraction by GC/MS shows the presence of hydroxyproline, marker of collagen, in all three samples. The amino acid relative percentage contents of the painting samples (reported in Table 6) was subjected to a multivariate statistical analysis together with a data set of 121 reference samples of animal glue, egg and casein, using the principal components analysis (PCA) method [32] .

**Table 6:** Amino acidic relative percentage contents and characteristic ratio values of the fatty acids of samples VIC2, VIC5 and VIC7

**Sample**	**Amino acidic relative percentage content**											**A/P**	**P/S**	**ΣD (%)**
	**Ala**	**Gly**	**Val**	**Leu**	**Ile**	**Ser**	**Pro**	**Phe**	**Asp**	**Glu**	**Hyp**			
VIC 2	8.1	21.3	5.0	9.3	4.5	8.6	2.0	3.8	18.6	18.4	0.4	1.4	1.3	48.9
VIC 5	15.0	32.3	4.8	6.8	2.9	7.1	11.4	2.0	10.2	1.0	6.6	0.5	1.6	23.4
VIC 7	12.2	21.6	4.5	6.1	3.2	4.8	10.6	3.5	9.5	12.8	11.4	6.0	1.1	79.7

**Figure 5:**
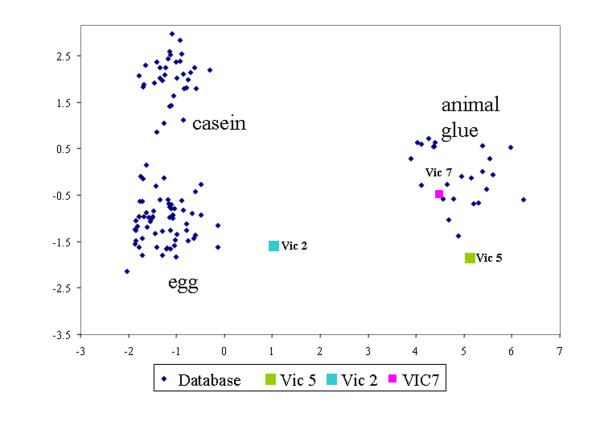
Principal Component analysis score plot of the amino acids percentage relative content in samples VIC2, VIC 5 and VIC7.

The PCA score plot (Figure 5) shows that VIC 7 and VIC 5 are located in the animal glue cluster or close to it. However, VIC 5 shows a percentage content of glutammic acid (Table 6) quite low for animal glue, this might be the result of a bacterial attack of the painting [33,34]. This ascertained degradation process does not allow us to exclude that another proteinaceous materials was simultaneously present. The presence of, hexadecanonitrile, marker of egg, in the pyrogram of this sample, allows to conclude that both egg and animal glue are present in VIC 5. VIC 2 contains both egg and animal glue, as it can be assessed from its position in the PCA score plot [5].

**Figure 6:**
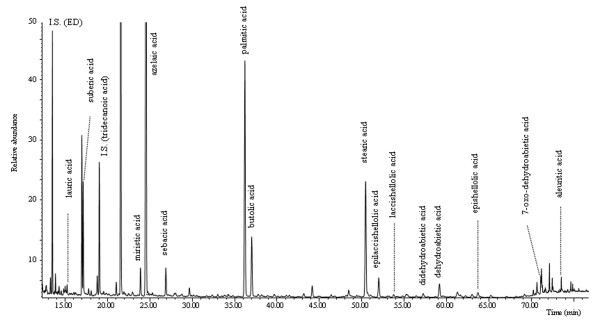
Total ion chromatogram of sample VIC 2 obtained by GC/MS procedure. Legend: Identified peaks are labelled.

The most abundant peaks in all chromatogram of the lipidic fraction (presented in Figure 6) are palmitic, stearic, suberic, azelaic, and sebacic acids. The calculated characteristic parameters for these acids [1] are reported in Table 6.

The A/P ratio for samples VIC 2 and VIC 7 clearly points out to a drying oil. As the A/P and P/S ratios obtained for sample VIC 2 are perfectly in agreement with those of a reference linseed oil, it can be concluded that egg, which was detected by the analysis of the proteinaceous fraction, is a minor component For VIC 5, the A/P ratio lower than 1 seems to point to a mixture of a drying oil (attested by a consistent amount of dicarboxylic acids) with a non drying fat. The presence of egg has been already evidenced by pyrolisis (see above).

Finally, in the acidic fraction of VIC 2 and VIC 7 the presence of the molecular markers of a Pinaceae resin (dehydroabietic acid together with didehydroabietic and 7-oxo dehydroabietic acid) and shellac (butolic, aleuritic, epishellolic, shellolic, laccishellolic, epilaccishellolic acids) were individuated [6,32,35-37].

### Synchrotron radiation micro FTIR

A critical point in performing SR FTIR mappings in transmission mode is the sample preparation as it is necessary to obtain very thin sample sections to avoid the complete absorption of the transmitted beam. Microtoming after embedding the sample in a epoxy resin usually causes several problems such as infiltration of the resin and crumbling and lost of particles [3,4]. Notwithstanding this, an epoxy resin worked particularly well with VIC samples given their high content in organic materials, resulting in flexible intact cross sections, which did not show any contamination of the embedding resin and were easy to cut.

**Figure 7:**
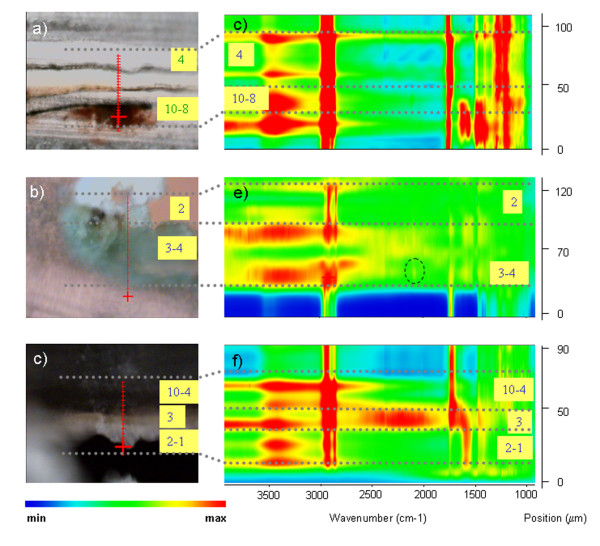
**Photomicrograph of the microtomed cross-sections. a)** VIC 2(12 μm); **b)** VIC 5(4 μm); **c)** VIC 7 (12 μm); line scan of **d)** VIC 2; **e)** VIC 5; **f)** VIC 7. The red line marks the line selected to perform the line scan. Squared numbers indicate the layers from the cross-section of the sample. Grey dotted lines indicated the position (in both the linescan and the stratigraphy) in which spectra features change.

A photomicrograph of each cross-section of the samples after microtoming is shown in Figure 7 (a, b, c). The thin section of VIC 2 contained only superficial layers (10 to 4).

**Figure 8:**
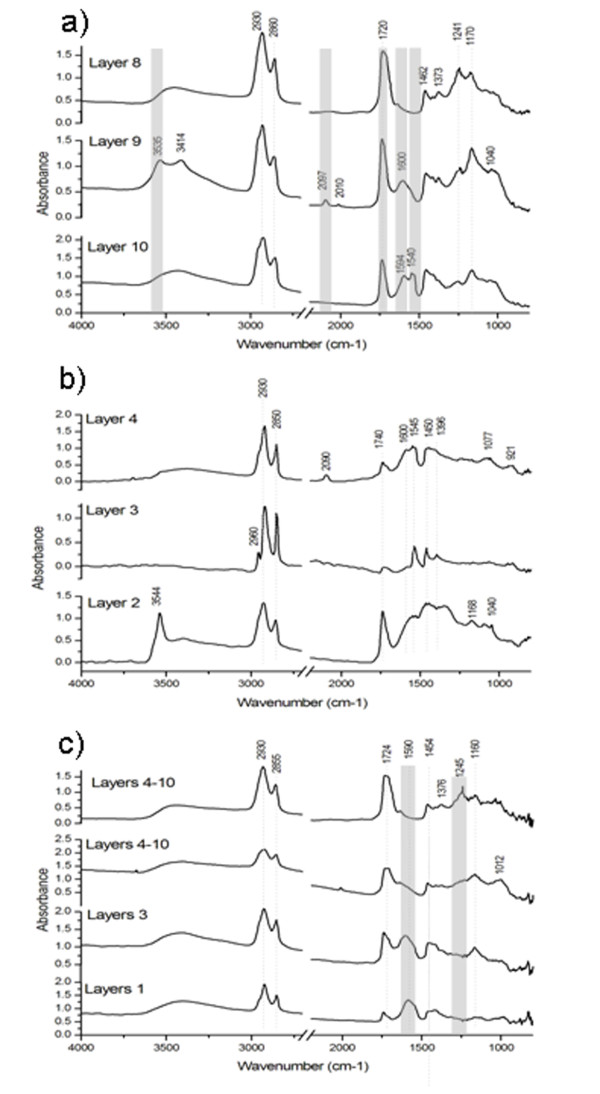
**Characteristic SR FTIR spectra of some layers of the samples a) VIC 2; b) VIC 5; c) VIC 7.** Spectra have been recorded with a 8x8 μm² aperture, 50 scans, 4 μm step and 8 cm^-1^ resolution. The bands chosen for the mapping are highlighted in grey.

The linescan permits to easily visualise the distribution of the main functional groups in the spectra along the line depicted in the photomicrograph of the sample (Figure 7 a,b,c). The linescan, being a representation of the spectra acquired at a sequence of points (wavenumbers vs position), permits to visualise the main spectral features (red color) of the different layers. The features highlighted in the linescans, mainly correspond to CH st (2800–3000 cm^-1^), carbonyl bands (1650–1750 cm^-1^), CO st (1175 cm^-1^ for oils and 1260 cm^-1^ for resins) and metal carboxylates bands (1600–1500 cm^-1^). In Figure 8, some representative SR FTIR spectra of each sample are presented.

In Table 7 the main spectral features (wavenumbers), highlighted by the linescan (red color) along the cross-section, are summarized. In this Table the linescan position between which those features are present is indicated and this information is related to the layer in the cross-section. The organic material identified in the spectra are also specified.

**Table 7:** Summary of the information obtained from the linescan and the spectra

**Sample**	**Layer**	**Linescan position (μm)**	**Linescan main features (cm^-1^)**	**Other bands in the spectra (cm^-1^)**	**Material identified from the spectra**	**Mapped bands (cm^-1^)**
VIC 2	10-9	10-45	1590 1530 1410 (ba) 1167	2930, 2850, 1730, 1590, 1530, 1462, 1415, 1387, 1240, 1167, 1040	Oil	1590 1540
	9	40-45	-	2012	Bone black	-
			-	2092	Prussian blue	2092
	8-4	50-100	1710 1260 1160	2930, 2850, 1720, 1450, 1373, 1260, 1160	Resin	1715
VIC 5	3-4	30-90	-	2930, 2850, 1730, 1580,1540, 1460, 1415	Oil	-
	4	30-45	2090	2090	Prussian blue	-
			-	3699, 3620, 1100, 1050	Clay (kaolinite)	-
VIC 7	1-2	0-30	1580	2930, 2855, 1730, 1590, 1460, 1380,	Oil	1580
	3	30-45	1580-1730 (broad area)	2930, 2855, 1730, 1590, 1460, 1387, 1160	Oil	-
	4-10	45-65	-	3535, 1410	Lead white	-
			-	2012	Bone black	-
			1730	2930, 2855, 1720, 1632, 1460, 1376, 1245, 1160	Resin	1250

**Figure 9:**
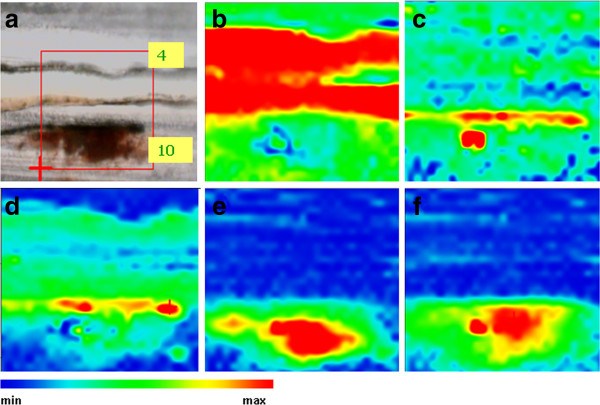
**a) Photomicrograph of the microtomed cross-section of VIC 2 (width: 12 μm).** The rectangle marks the area selected to perform the SR FTIR mapping; chemical image of **b)** 1717, **c)** 2090, **d)** 3539, **e)** 1590 and **f)** 1533 cm^-1^. Mapped area 102 x 174 μm.

**Figure 10:**
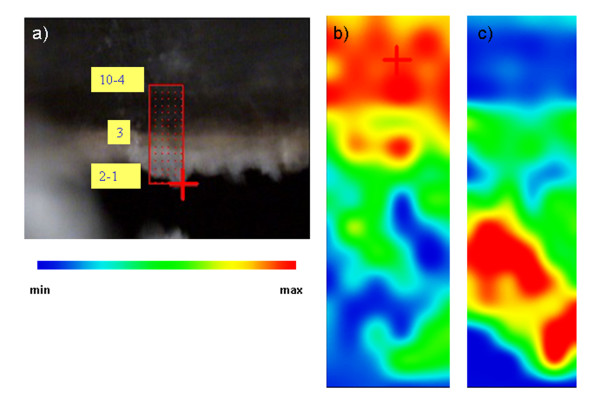
**a) Photomicrograph of the microtomed cross-section of VIC 7(12 μm). chemical images of b) 1250 cm**^**-1 **^**and c) 1590 cm**^**-1**^**.** Mapped area is 131 x 48 μm. The rectangle in a) marks the area selected to perform the SR FTIR mapping.

By mapping the bands specified in Table 7 the distribution of some of the organic materials could be achieved for VIC 2 and VIC 7. This was not possible for sample VIC 5 due to irregularities in the width of the slice. False color maps are shown in Figures 9 and 10 for VIC 2 and VIC 7, respectively, and represent the distribution of specific functional group (color is a function of the peak height versus position) in the cross-section. Mappings resulted from the accurate study of individual spectra to assure that the highlighted areas were consistent with the material localization.

SR micro FTIR mapping of the organic materials identified by GC/MS highlights the alternate use of oil, used as binding medium of the thin pigment layers, and resin layers. By mapping the bands at 1715 and 1260 cm^-1^, considered as characteristic of a terpenoid resin, wide areas presenting the maximum intensities are coincident with the non pigmented layers on top of the stratigraphies. By mapping the carboxylate peaks (1600–1540 cm^-1^), the presence of the saponified oil in the pigmented layers is also established. Finally, mapping of the bands at 2092 cm^-1^ and 3535 cm^-1^ allowed establishing the distribution of Prussian blue in layer 8 and zinc white in layers 10 to 8 in VIC 2.

### Synchrotron radiation XRD

**Figure 11:**
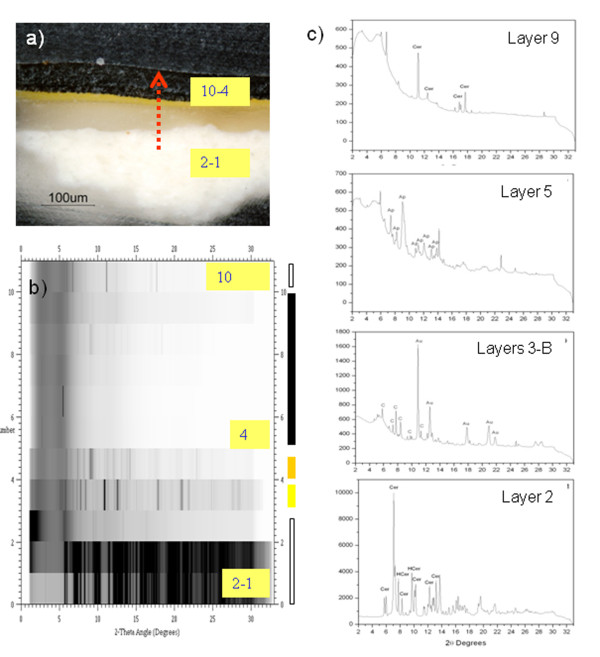
**a) polished cross-section prepared for XRD analysis (the arrow indicated the linescan position and its direction) b) XRD linescan from 0 (corresponding to layer 1–2) to 13 (corresponding to layer 10); c) XRD patterns of some of the scans corresponding to different layers.** Peaks labeled with letters corresponding to: * (cerussite, PbCO_3_), Ap (Hydroxyapatite, Ca_5_(PO_4_)_3_(OH)), Au (gold), C(crocoite, PbCrO_4_) and HCer (hydrocerussite, Pb_3_(CO_3_)_2_(OH)_2_).

Sample VIC 7 was prepared for micro XRD experiments in transmission mode performing line scan measurements across the sample cross-sections. Figure 11 shows the 2θ diffraction peaks in respect to vertical position on the chromatic layers: the alternation of organic (non diffracting amorphous layers corresponding to the white areas) and crystalline phases (with the high intensity diffraction peaks corresponding to the grey and black areas) is easily appreciated. The line scan is acquired in the area of the cross section evidenced by a red line in the cross section image reported in Figure 11a. XRD patterns from the most characteristic chromatic layers are also presented (Figure 11c).

It is important to specify that the pattern matching process presented some difficulties due to the nature of the samples: painting cross-sections are formed by a mixture of different crystals of different size and orientation that are not powdered to obtain a statistically arranged mixture of crystals, all the more so when analyzed with a micrometric beam. Samples are not, thus, presenting all possible orientation under the excitation rays. Though some crystals are small enough to be considered randomly oriented, big crystals such as cerussite and hydrocerussite are bigger than the beam spot used obtaining a diffraction pattern different from that of the standards and thus difficult to be identified. However, SR XRD permitted the unequivocal characterization and layer location of most of the pigments and dryers (barite (BaSO_4_), hydroxyapatite (Ca_5_(PO_4_)_3_OH), cerussite (PbCO_3_), hydrocerussite (Pb_3_(CO_3_)_2_(OH)_2_), crocoite (PbCrO_4_)) present (Table 3). It is interesting to note the identification of lead acetate (Pb(C_2_H_3_O_2_)_2_ . 3H_2_O), a dryer for oil paints, in sample VIC 7 [38]. To the best of our knowledge this is the first experimental evidence of the use of lead acetate as dryer in painting samples.

## Discussion

The multi analytical approach used allowed us to gain many pieces of information about the samples and the combined interpretation of the data obtained with different techniques permitted the characterization of the samples layer by layer. Table 8 reports the organic materials characterised as well as their distribution in relation with inorganic ones (from Table 3).

**Table 8:** Materials identified with the different techniques

**Sample**	**Layer number**	**FTIR**	**Py/GC/MS**	**GC/MS**	**SR FTIR**	**Organic compound identified**	**Inorganic materials (from table**8**)**
VIC 2	10	Natural resin Lipid material	Drying oil Pinaceae resin Shellac Polysaccaridic material	Linseed oil Pinaceae resin Shellac Egg Animal glue	Lipid material	Linseed oil	Iron oxide Calcite Zinc compound Calcium oxalates Quartz
	9				Lipid material	Linseed oil	Bone black Prussian blue
	8				Natural Resin	Shellac, pine resin	-
	7				Natural Resin	Shellac, pine resin	-
	6						
	5						
	A	-			-		Gold leave
	4	-			Natural Resin	Shellac, pine resin	
	B				-	-	Brass leave
	3	Lipid material			Lipid material	Linseed oil	Barium sulphate Lead chrome yellow
	2				Lipid material	Linseed oil	
	1				Lipid material	Linseed oil	Clay? Iron oxide?
	C				-	-	Brass leave
VIC 5	4	Lipid material	Drying oil Polysaccharidic material Egg (traces)	Drying oil Non drying fat (egg?) Animal glue	Lipid material	Linseed oil, egg	Strontium yellow Prussian blue Lead white Kaolinite zinc compound
	3				Lipid material	Linseed oil, egg	
	2	Lipid material			Lipid material	Linseed oil	Lead white
	1	Polysaccharide material				Mixture of polysaccharide materials	
VIC 7	10	Natural resin Polysaccharide material	Drying oil Pinaceae resin Shellac Polysaccharidic material	Prepolymerized linseed oil Pine resin Shellac Animal glue	Resin	Shellac	
	9				-	-	Bone black Prussian blue
	8				Resin	Shellac, pine resin	
	7				-	-	-
	6				resin	Shellac, pine resin	
	5				Proteinaceous material	Animal glue	Bone black
	4				Resin		
	A				-	-	Chrome yellow Gold
	3	Lipid material			Lipid material	Linseed oil	Lead white Zinc white
	2	Lipid material			Lipid material	Linseed oil	Zinc white
	1				Lipid material	Linseed oil	Lead white Lead acetate

Pigments identified (see also Additional file 1) are mainly of natural origin (such as green earth, calcite or bone black) though some synthetic materials from the end of the 19^th^ century such as chrome green or strontium yellow were also used by the painter. In sample VIC 5, “green cinnabar”, a mixture of Strontium yellow and Prussian blue has been used. Lead white has been used in the preparation layers of samples VIC 5 and VIC 7.

A wide variety of metallic leaves have been used, some of them unusual: not only gold and silver ones but also alloys of Ag/Au and Cu/Zn or Al/Pd (see Additional file 1) depending on the in-depth in the sample of those metallic leaves and the effect to be produced.

As far as the organic materials are concerned, a drying oil has been used as binding medium in pigment layers as well as in the mordants of the metallic leaves, while proteinaceous materials (egg and animal glue) were mainly used for canvas preparation layers. In some particular samples, proteinaceous materials were also used as binding media of pigment layers (such as VIC 5 and VIC 7). Polysaccharide materials were identified in all samples and localised in sample VIC 5 on the canvas preparation layer. Finally, natural resins (identified as shellac and a *Pinaceae* resin) were mainly localised in the superficial layers.

Metallic leaves have been applied in two different ways: on a linseed oil mordant or on a shellac layer. Moreover, different kinds of mordants have been also applied depending on the kind of metallic leaves and disposition in the stratigraphy. This way, VIC 2 and VIC 7 present a mordant made of linseed oil (used in paintings of big size due to its capacity of remain adherent for a long time) while other samples (results presented as Additional file 1) present linseed oil in mixture with lead white and Prussian blue, or a zinc containing material compounds (probably zinc white).

It is also of particular interest the widespread use of shellac (mixed with pine resin) to generate a high number of thin layers on top of the stratigraphy of VIC 2 and 7. Those layers alternated with pigment layers and metallic leaves generate the shiny silvery or gilded effect characteristic of the later painting technique of Josep Maria Sert. The fact that shellac was probably refined to painting purposes by using sodium hypochlorite explains the high concentrations of Cl (by EDS) in the shellac layers (Table 3). The mapping of Cl in the SEM polished cross-sections (results not shown) in fact was coincident with the non pigmented layers on top of the stratigraphy of those samples.

## Conclusions

The combined use of different techniques applied on different aliquots of the same sample, and the complementary interpretation of the results obtained, allowed us to establish the build-up of each sample, the materials used and, thus, to ascertain the painting technique of Josep Maria Sert in his paintings in the city of Vic and to appreciate his technical evolution. In particular synchrotron experiments gave the final answer to key questions allowing us to establish not ony the organic media distribution but also the presence of some pigments as well as the distribution of ageing products such as oxalates and carboxylates.

Josep Maria Sert works present two kinds of painting techniques. One is a traditional technique based on the application of one or few pigmented layers on a preparation, using mainly linseed oil as binding medium. The other technique is based on the application of several thin layers, mainly organic, with some thin pigmented layers and metallic leaves in the between. This study allowed to establish that Sert started using the first traditional technique, subsequently moving to the use of both techniques at the same time (painting “The four seasons” from 1917–1920) to finally use exclusively the complex multilayered painting technique in the last stage of his career. These experimental evidences correspond to the two different finishing described from art historians for Sert paintings: a polychrome and decorative mural painting and a monochrome painting (sepia, gilded and silvery tonalities) that was, in the end, assumed as his characteristic way of painting [17].

## Abbreviations

SEM-EDS: Scanning electron microscopy - Electron Dispersive Spectroscopy; SR μXRD: Synchrotron Radiation micro X-Ray Diffraction; μFTIR: micro Fourier Transform Infrared Spectroscopy; GC/MS: Gas Chromatography/Mass Spectrometry; PY/GC/MS: Pyrolisis/Gas Chromatography/Mass Spectrometry; PCA: Principal Component Analysis; HPLC: High Performance Liquid Chromatograpy; HMDS: Hexamethyldisilazane; MTBSTFA: *N*-*tert*-Butyldimethylsilyl-*N*-methyltrifluoroacetamide; IS: Internal Standard; ED: Hexadecane.

## Competing interest

The authors declare that they do not have competing interests.

## Authors’ contributions

All authors contributed to data analyses and to finalizing the manuscript. All authors have read and approved the final version.

## Authors’ information

**Anna Lluveras-Tenorio** majored in Chemical Science at the University of Barcelona (UB), Spain, in 2003. She worked as a research staff member at the University of Barcelona from 2003 until 2005. She obtained her PhD in July 2009 at the University of Barcelona. she currently holds a post-Doc Marie Curie position at the University of Pisa. Her research lines are the fundamental study of the organic materials used as binders as well as the development of analytical methodologies for painting analysis using GC-MS, FTIR, thermo gravimetric and synchrotron radiation based techniques.

**Alessia Andreotti** graduated in Chemistry in 2002 at the University of Pisa with a thesis on laser cleaning applied to the restoration of paintings. Since 2004, she has been working as a technician at the Department of Chemistry and Industrial Chemistry in the technical-scientific and data evaluation areas. Her research focuses on the characterization of natural and synthetic organic materials collected from samples in the field of Cultural Heritage using instrumental analytical techniques such as HPLC, GC/MS, Py-GC/MS, and direct exposure mass spectrometry (DE-MS). She also specializes in using lasers and other state-of-the-art techniques for cleaning of easel paintings, mural paintings, and other artifacts.

**Ilaria Bonaduce** is a lecturer and permanent researcher in the Department of Chemistry and Industrial Chemistry at the University of Pisa; she received her Ph.D. in Chemical Science from the University of Pisa, Italy, in 2006. Her research focuses on the characterization of natural and synthetic organic materials used in works of art and the study of how they degrade during aging. Another major research interest is the development of analytical procedures for the identification of organic materials in paint samples, using mass spectrometric techniques, such as GC/MS, Py-GC/MS, and DE-MS.

**Sarah Boularand** graduated in Chemistry at the University of Clermont-Ferrant and she is working in the cultural heritage field since 1999. Her research lines are the analysis of painting materials, both pigments and binders by means of spectroscopic and electron microscopic techniques.

**Marine Cotte** is beamline responsible at the micro-spectroscopy beamline (ID21), at the European Synchrotron Radiation Facility. Her researches are related to the analysis of ancient materials, in particular paintings, using the X-ray and a FTIR microscopes available at ID21.

**Josep Roqué** received a Ph.D. in Geology at the University of Barcelona (Spain) in 2007. He has been a post doctoral research associate at the Microfocus Spectroscopy beamline (I18) at the Diamond Light Source (UK) and at the Nanomateriaux Group at the CEMES-CNRS (Toulouse, France).

**Maria Perla Colombini** currently holds the post of Full Professor of Analytical Chemistry in the Department of Chemistry (Faculty of Science) at the University of Pisa. She holds courses on Analytical Chemistry and the Chemistry of Cultural Heritage. She is Director of the Masters Course on “Materials and Diagnostic Techniques in the Cultural Heritage field”. Her research work includes developing analytical procedures based on spectroscopic and chromatographic techniques for characterizing micropollutants in the environment and, especially, organic materials and their degradation products in works of art and archaeological objects. She is head of the Chemical Sciences for the Safeguard of Cultural Heritage research group and specializes in the characterization of binders, organic dyes, and resins using chromatographic and mass-spectrometric techniques.

**Marius Vendrell-Saz** received a Ph.D. in Geology from the University of Barcelona (Spain) and is currently Associated Professor of Cristallography at the University of Barcelona. Since 1984 his research has been focused on the study of the materials used in the cultural heritage, from building materials to paintings.

## Supplementary Material

Additional file 1 **Table S1.** Summary of the morphological characterization of the samples. **Table S2**. FTIR and Py/GC/MS results for the 4 paint samples. Information on the layers is given when possible. Click here for file
