# Supplementary material for: Mass Spectrometric and Synchrotron Radiation based techniques for the identification and distribution of painting materials in samples from paints of Josep Maria Sert
Source: Chem Cent J. 2012 May 22;6:45. doi: 10.1186/1752-153X-6-45 (PMC3505478; doi:10.1186/1752-153X-6-45)
Supplement: Additional file 1 — Table S1. Summary of the morphological characterization of the samples. Table S2. FTIR and Py/GC/MS results for the 4 paint samples. Information on the layers is given when possible. [file 1752-153X-6-45-S1.doc]

The following additional data are available with the online version of this paper. The supplementary material shows a summary of the results obtained from the characterization of the materials present in other 4 samples from Josep Maria Sert studied at the same time with the 3 samples showed in the paper. The results by conventional FTIR and Py/GC/MS complement the results obtained in the paper and help the discussion on the painting technique of Sert.

**Supplementary data**

SP 1. Summary of the morphological characterization of the samples.

| Sample | Appearance of the surface | Total Number of layers | Thickness  (min-max /µm) | Number of highly pigmented layers | Number of mainly organic layers | Number of metallic layers | Metallic leaves |
| --- | --- | --- | --- | --- | --- | --- | --- |
| VIC 1 | Dark blue | 9 | 1-50 | 2 | 5 | 2 | Au  Cu-Zn |
| VIC 3 | Silvery | 13 | 25-150 | 6 | 5 | 2 | Au-Ag |
| VIC 4 | Dark green | 5 |  | 4 | 1 | 0 | - |
| VIC 6 | Black - golden | 10 | 2-200 | 5 | 3 | 2 | Al-Pd  Cu-Zn |

SP 2. FTIR and Py/GC/MS results for the 4 paint samples. Information on the layers is given when possible.

| Sample | Layer | FTIR | | Py/GC/MS |
| --- | --- | --- | --- | --- |
|  |  | Organic material | Inorganic materials |  |
| VIC 1 | Superficial  layers | Natural resin | Ultramarine blue | Drying oil  Pinaceae resin  Shellac  Saccharide material |
|  | Between metallic leaves | Natural resin - | - |  |
| VIC 3 | Superficial organic layers | Natural resin | Calcium carbonate  Barium sulphate | Drying oil  Pinaceae resin  Shellac  Saccharide material |
|  | Between metallic leaves | Natural resin | - |  |
|  | Under metallic leave | Lipid material | Prussian blue  Lead white |  |
|  | Preparation | Lipid material | Calcium carbonate  Barium sulphate  Lead white |  |
| VIC 4 | Pigment layers | Lipid material | Green Earth  Bone black  Barium sulphate  Calcium carbonate | Drying oil  Saccharide material |
|  | Preparation | Lipid material | Calcium carbonate  Barium sulphate  Quartz |  |
| VIC 6 | Superficial layers | Natural resin | Bone black | Drying oil  Pinaceae resin  Shellac  Saccharide material |
|  | Mordent | Lipid material | Calcium carbonate |  |
|  | Preparation | Lipid material | Calcium carbonate  Lead white |  |
